# Review on Non-Volatile Memory with High*-k* Dielectrics: Flash for Generation Beyond 32 nm

**DOI:** 10.3390/ma7075117

**Published:** 2014-07-15

**Authors:** Chun Zhao, Ce Zhou Zhao, Stephen Taylor, Paul R. Chalker

**Affiliations:** 1 Department of Electrical Engineering and Electronics, University of Liverpool, Liverpool L69 3GJ, UK; E-Mails: chun.zhao@liverpool.ac.uk (C.Z.); s.taylor@liverpool.ac.uk (S.T.); 2 Department of Electrical and Electronic Engineering, Xi’an Jiaotong-Liverpool University, Suzhou 215123, China; 3 Department of Materials Science and Engineering, University of Liverpool, Liverpool L69 3GH, UK; E-Mail: pchalker@liverpool.ac.uk

**Keywords:** non-volatile memory, flash, high*-k* dielectrics, charge trapping memory

## Abstract

Flash memory is the most widely used non-volatile memory device nowadays. In order to keep up with the demand for increased memory capacities, flash memory has been continuously scaled to smaller and smaller dimensions. The main benefits of down-scaling cell size and increasing integration are that they enable lower manufacturing cost as well as higher performance. Charge trapping memory is regarded as one of the most promising flash memory technologies as further down-scaling continues. In addition, more and more exploration is investigated with high*-k* dielectrics implemented in the charge trapping memory. The paper reviews the advanced research status concerning charge trapping memory with high*-k* dielectrics for the performance improvement. Application of high*-k* dielectric as charge trapping layer, blocking layer, and tunneling layer is comprehensively discussed accordingly.

## 1. Introduction

A non-volatile memory device is one that can retain stored information in the absence of power and flash memory is a type of non-volatile memory [1]. Floating-gate flash memory has been successfully developed in the last few decades with continues down-scaling the dimensions of the cell to obtain high data-storage density, high program/erase speeds, low operating voltage and low power consumption [2]. The ever-increasing fabrication density of Flash memory has been mainly driven by area scaling [3]. However, some intrinsic limitations make this type of memory rapidly approach the scaling limit. As Flash memory device scales down beyond the 32 nm technology node, approaches face significant challenges. A relatively thick tunneling oxide and inter poly dielectric layer have to be used in the floating-gate memory to maintain acceptable reliability, limiting further down-scaling of the cell size in the vertical direction [4]. In addition, maintaining a high gate coupling ratio is still one main bottle-neck for down-scaling the floating-gate devices [5]. Moreover, as the spacing between adjacent devices is down-scaled, this parasitic capacitance plays an increasingly dominant role in the device performance due to data stored in the adjacent cells can interfere with each other by capacitive coupling [6]. Additionally, a limited margin poses a great challenge on the reliability of the floating-gate memory devices, as the number of electrons stored in floating-gate significantly decreases with continual down-scaling of the cell size [7].

Some promising memory technologies have been developed for the next-generation flash memory to go beyond the current floating-gate flash memory technology. The ferroelectric field effect transistor (FeFET) is a one transistor (1T) memory device where a ferroelectric capacitor is integrated into the gate stack of a FET [8]. The ferroelectric polarization directly affects charges in the channel and leads to a defined shift of the output characteristics of the FET. At the channel interface, a high quality insulator is required to guarantee a low interface state density. For this reason it is very difficult to fabricate a FeFET with excellent electrical properties. Scaling is projected to end approximately with the 22 nm generation, because the insulation layer becomes too thin and the properties of the ferroelectric with respect to thickness dependence of the coercive field will not allow further reduction [9]. In addition, the major challenge is the long-term reliability related to the ferroelectric-semiconductor interface. Another important challenge is the rather short retention time, approximately 30 days, for the FeFET. The nanoelectromechanical memory (NEMM) is based on a bi-stable nano-electromechanical switch [10]. In this concept, mechanical digital signals are represented by displacements of solid nanoelements, which result in closing or opening an electrical circuit. Several different modifications of suspended-beam NEMMs are currently being explored using different materials including carbon nanotubes, Si, Ge, and TiN. A difficult challenge of the suspended-beam NEMM is scalabily: according to a recent study, it might be difficult to achieve low-voltage (~1 V) operation for the beam length less than 50 nm [11]. The spin transfer torque memory (STTRAM) is an advanced version of the magnetic RAM with a different write mechanism [12]. The memory cell consists of a semiconductor isolation device and a magnetic tunnel junction with two ferromagnetic layers separated by a MgO-based tunneling barrier layer in which thickness is controlled to approximately 1 nm. Key factors in STTRAM development include reducing STT writing current and voltage (energy) while maintaining adequate thermal stability. In nanothermal memory elements, consisting of a nano-scale metal-insulator-metal (MIM) structure, typical resistive switching phenomena are based on thermal effects, which result in unipolar switching characteristics [13]. The other type of nanothermal memory is nanowire-structured phase change cell, for which the underlying phase transformation between amorphous and crystalline phases is similar to the conventional phase-change memory (PCM) [14]. Compared to prototypical PCM, the switching current and therefore the write energy could be considerably reduced in nanowire-PCM cells. The principle challenge is fabrication of the nanowire crossbar memory cells containing the required select diodes, perhaps using self-assembly or directed-assembly technology. The nanoionic memory operation is based on a change in resistance of a MIM structure caused by ion (cation or anion) migration combined with redox processes involving the electrode material or the insulator material, or both [15]. Precise predictions are not yet possible, because many details of the mechanism of the reported phenomena are still unknown. Developing an understanding of the physical mechanisms governing switching of the nanoionic memory is a key challenge for this technology. Macromolecular memory sometimes referred to as polymer or organic memory consists of a memory element, which is a thin-film of organic material [16]. It is, in some cases, similar to molecular memory, but extreme scaling is not important, while reduced fabrication cost is emphasized. Charge-trapping memory (CTM) was firstly introduced in 1967 to show some distinguished advantages over the conventional floating-gate counterpart [17]. It defines the program/erase states via adding charges to and removing charges from the charge-storage layer respectively, similar to floating-gate memory. Apart from the floating-gate cell where charges are mainly stored in the conduction band of the floating gate, the main difference of charge-trapping flash memory is that charges are located at the spatially discrete traps distributed in the band-gap of the charge-trapping layer. CTM is totally compatible with the complementary metal-oxide-semiconductor (CMOS) technology, and easy to be integrated with current fabrication process.

NOR-type CTM flash memories were the first to be introduced towards the scaling for two decades. Until recently, non-volatile memory half-pitches have lagged behind those for dynamic random access memory (DRAM) or CMOS logic devices in the same year [18]. Rapid progress in NAND CTM flash technology has not only reversed this trend, but also surpassed the half-pitches of DRAM and CMOS logic devices. NAND flash memories have seen tremendous demand in this decade. Both NOR and NAND flash memories are now facing major roadblocks in continued scaling. NOR flash memory devices are programmed by channel hot-electron injection. Since the silicon-oxide barrier height is 3.2 eV, the drain voltage has to be at least greater than 3.2 V for reasonable efficiency. Therefore, there is a major challenge in scaling the drain voltage in NOR flash devices [19]. The scaling of tunnel oxide is limited by concerns for reliability issues. As NOR flash memories provide direct cell access, the reliability issues are more stringent, unlike NAND flash memories which can use error code correction and data re-mapping strategies. The tunnel oxide thickness for NOR flash devices is essentially stuck at 8–9 nm and not scaled anymore. There are a number of factors contributing to difficulty in scaling channel length in NOR flash devices. The tunnel oxide does not scale anymore, because a thinner tunnel oxide reduces short-channel effects. Hence, the channel length faces potentially game-ending scaling issues and consequently, so does the cell area. As for NAND flash memory, the scaling of high-density is limited by parasitic interference between adjacent cells since they are extremely close to each other. The charge trapping layer (CTL), being a capacitive-coupled electrode, has started to have significant coupling with the charge trapping layer of adjacent cells and other electrodes of neighboring cells. This causes an undesirable shift in the state of one cell due to neighboring cells. Like NOR flash, tunnel oxide scaling in NAND flash is also limited by reliability concerns. The tunnel oxide thickness is stuck at ~6 nm and needs breakthroughs to continue scaling beyond that. The control dielectric thickness is also limited by reliability concerns, similar to the tunnel oxide. Reliability concerns are also very serious if the control dielectric is modified to use high-*k* dielectrics, which may be necessary to improve gate coupling ratio. The ultimate intrinsic limits of NAND flash memories are likely to be due to statistical fluctuations induced by too few electrons stored. The International Technology Roadmap for Semiconductors (ITRS) scaling projection for floating-gate NOR and NAND flash is shown comprehensively in the following tables (Table 1 and Table 2). Nowadays, high*-k* dielectrics [20,21,22,23,24,25,26,27,28,29,30,31,32] are highly considered and widely implemented for CTM upon continually scaling down of the dimensions of flash memory [33]. The advantage of using high*-k* dielectrics is that for the same equivalent oxide thickness (EOT), the high*-k* dielectrics can have a thicker physical thickness than silicon dioxides. The using of high*-k* dielectrics makes it possible for continual down-scaling of the cell size.

In the paper, the CTM with high-*k* dielectrics are reviewed. Firstly, the characterization of CTM is introduced. Then, concerning high*-k* dielectrics as the core of CTM: charge trapping layer, much more efforts are focused within the paper. Afterwards, the paper briefly reviews some high*-k* dielectric application as blocking and tunneling layer associated with CTM for alternative option to replace thermal oxides, despite reliability concerns might be a very serious issue if the tunneling dielectric is modified to use high-*k* dielectrics. Finally, conclusion in the end gives a summary of this research work, also provides a research direction for the next-generation flash memory.

**Table 1:** International Technology Roadmap for Semiconductors (ITRS) Scaling projections for floating-gate NOR flash [18].

NOR flash	2009	2010	2011	2012	2013	2014
NOR flash technology node—*F* (nm) [21]	50	45	40	35	32	28
A. floating gate NOR flash						
Cell size-area factor in a multiples of *F*^2^ [22,23,24,25]	9–11	9–11	9–11	9–11	9–11	9–11
Gate length *L*_g_, physical (nm) [26]	110	110	100	100	90	90
Tunnel oxide thickness (nm) [27]	8–9	8–9	8–9	8–9	8	8
Interpoly dielectric material [28]	ONO	ONO	ONO	ONO	High-*k*	High-*k*
Interpoly dielectric thickness EOT (nm)	13–15	13–15	13–15	13–15	8–10	8–10
Gate coupling ratio [29]	0.6–0.7	0.6–0.7	0.6–0.7	0.6–0.7	0.6–0.7	0.6–0.7
Highest W/E voltage (V) [30]	7–9	7–9	7–9	7–9	6–8	6–8
*I*_read_ (μA) [31]	21–27	20–26	19–25	17–22	15–20	14–19
Endurance (erase/write cycles) [32]	1.0 × 10^5^	1.0 × 10^5^	1.0 × 10^5^	1.0 × 10^5^	1.0 × 10^6^	1.0 × 10^6^
Nonvolatile date retention (years) [33]	10–20	10–20	10–20	10–20	20	20
Maximum number of bits per cell (MLC) [34]	2	2	2	2	2	2
Array architecture (with cell contact (CC) or virtual ground (VG)) [35]	CC	CC	CC	CC	CC/VG	CC/VG

**Table 2:** ITRS Scaling projections for floating-gate NAND Flash [18].

Year of production	2009	2010	2011	2012	2013	2014
DRAM 1/2 pitch (nm) (contacted)	50	45	40	35	32	28
MPU/ASIC metal I (MI) 1/2 PITCH (nm) contacted	54	45	38	32	27	24
(ORTC) NAND flash poly 1/2 pitch (nm)	38	32	28	25	23	20
(PIDS) NAND flash poly 1/2 pitch (nm)	34	32	28	25	22	20
NAND flash						
NAND flash technology node—*F* (nm) [1]	34	32	28	25	22	20
Number of word lines in one NAND string [2]	64	64	64	64	64	64
Cell type (FG, CT, 3D, *etc.*) [3]	FG	FG	FG	FG/CT	FG/CT	CT/3D
3D NAND number of memory layers	1	1	1	1	1	2
A. Floating gate NAND flash						
Cell size-area factor in a multiples of *F*^2^ SLC/MLC [4]	4.0/1.3	4.0/1.3	4.0/1.3	4.0/1.0	4.0/1.0	4.0/1.0
Tunnel oxide thickness (nm) [5]	6–7	6–7	6–7	6–7	6–7	6–7
Interpoly dielectric material [6]	ONO	ONO	ONO	High-*k*	High-*k*	High-*k*
Interpoly dielectric thickness (nm)	10–13	10–13	10–13	9–10	9–10	9–10
Gate coupling ratio (GCR) [7]	0.6–0.7	0.6–0.7	0.6–0.7	0.6–0.7	0.6–0.7	0.6–0.7
Control gate material [8]	*n*-Poly	*n*-Poly	*n*-Poly	Poly/metal	Poly/metal	Poly/metal
Highest W/E voltage (V) [9]	17–19	17–19	17–19	15–17	15–17	15–17
Endurance (erase/write cycles) [10]	1.0 × 10^5^	1.0 × 10^5^	1.0 × 10^5^	1.0 × 10^4^	1.0 × 10^4^	1.0 × 10^4^
Nonvolatile date retention (years) [11]	10–20	10–20	10–20	10–20	10–20	20
Maximum number of bits per cell (MLC) [12]	3	3	3	4	4	4

## 2. Background Knowledge

**Figure 1:**
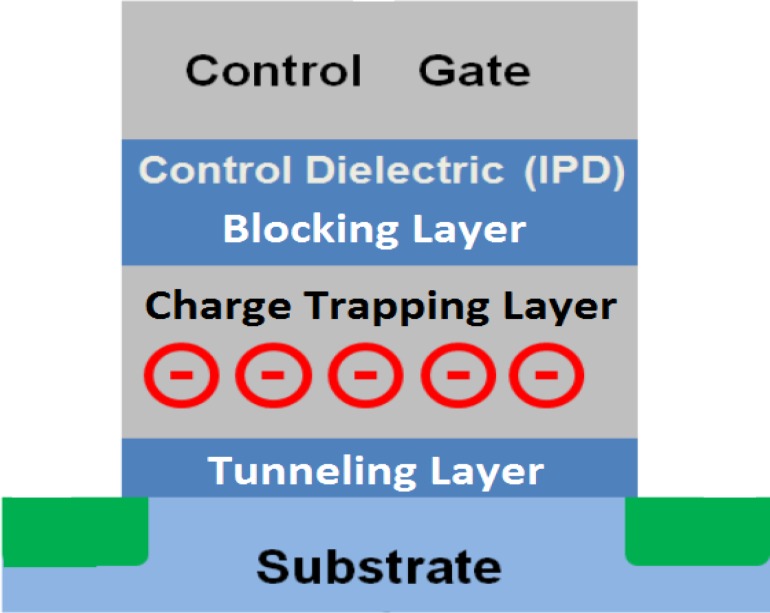
Cross-section schematic of a charge trapping memory transistor.

The structure of a charge trapping memory transistor is similar to that of a regular metal oxide silicon (MOS) transistor, except for an additional dielectric layer (charge trapping layer) between the blocking layer and the tunneling layer. Figure 1 shows the cross-section schematic. The charge-trapping layer is electrically isolated from surrounding layers on all sides by dielectrics. The dielectric layer between the charge trapping layer and the gate is called the blocking layer or inter-poly dielectric (IPD) because both of the gate and IPD are usually made of poly-silicon originally. The dielectric closest to the substrate is called the tunnel layer. The name originates from the working principle that the erase operation and the program operation injects through this thin oxide via quantum mechanical tunneling.

**Figure 2:**
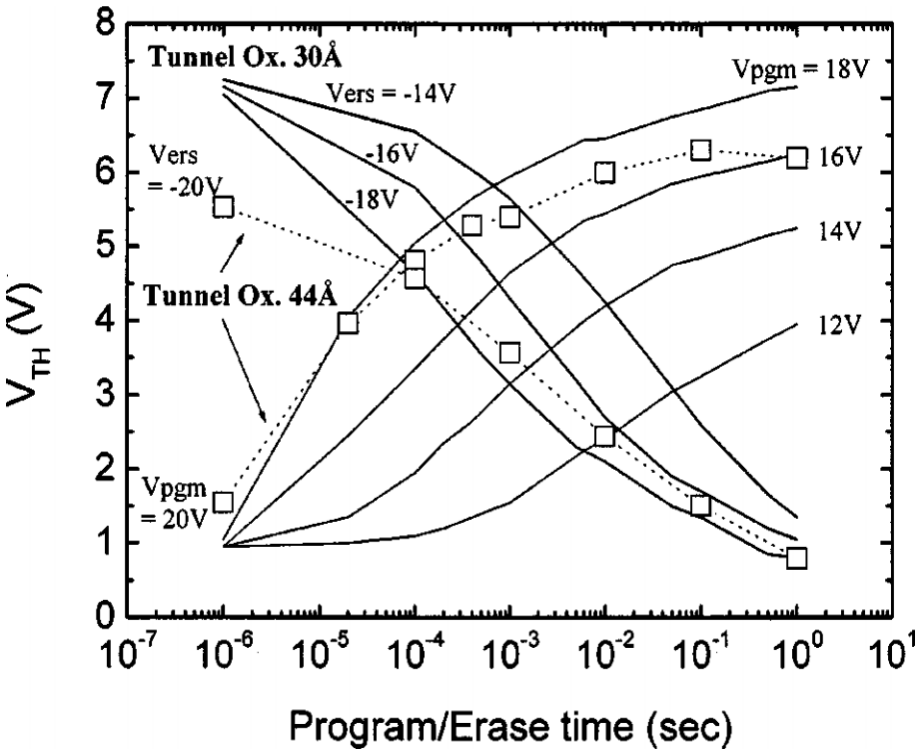
Program and erase characteristics of SANOS devices. Reused with permission from [34], Copyright 1997, IEEE.

As the name indicates, the charge-trapping layer serves as the charge storage dielectric. Charge-trapping type flash memory determines the digital “1” and “0” by charges insertion and removal from the charge-trapping layer, which can be considered as program/erase processes respectively. The program/erase speed is usually defined as variation of threshold voltage with respect to time. Figure 2 presents the program/erase speed of one kind of CTMs. For instance, concerning the program mode, a positive pulse is applied to the gate, causing electrons to be injected from the substrate into the charge-trapping layer. The stored electrons lead to a positive shift of threshold voltage. In the erase mode, a negative pulse is applied to the gate in order to cause holes to be injected from the substrate into the CTL, and/or electrons escaping from the CTL into the substrate, which causes a negative shift of threshold voltage. Obviously, a low operating voltage with a short pulse-width (prompt program/erase speed) is desirable for memory devices nowadays. A blocking layer is used in flash memories. The thickness heavily influences program/erase speed and the magnitude of read current for an industry-standard flash cell. Low defect density and long mean time to failure, together with charge retention capability, are important reliability issues.

Programming is obtained by applying pulses to the control gate and to the drain simultaneously when the source is grounded. This operation can be performed selectively by applying the pulse to the word line (WL), which connects the control gates, and biasing the bit line (BL), which connects the drains [34]. The change in threshold voltage depends upon the width of the programming pulse. For some cases, to have a voltage shift of around 3, 3.5 V, a pulse width with typical values in the 1–10 us range must be applied. A rapid change in cell *V*_T_ occurs initially. Then, as the charge trapping layer potential drops below the drain potential, *V*_T_ saturates. At this point, we can define an intrinsic threshold. The electric field in the tunnel oxide close to the drain reverses and electron injection into the charge-trapping layer is much less favorable [35]. Intrinsic threshold voltage shift, roughly, does not depend on the channel length but depends on the coupling ratios (the overlap between the charge trapping layer and control gate on field oxide). In addition, intrinsic threshold voltage shift also depends linearly on drain voltage. Temperature also has an influence on programming speed. A higher temperature reduces the number of hot electrons available for injection, hence retarding the programming characteristics.

The erase operation requires a high voltage pulse to be applied to the source (common to all the transistors in the array/block) when control gates (WL) are grounded and drains (BL) floating. Before applying the erase pulse, all the cells in the array/block are programmed to start with all the thresholds approximately at the same value [34]. After that, an erase pulse having a controlled width is applied. Electrical erase is achieved via tunneling of charge from the charge-trapping layer to the source. To have a junction that can sustain the high, applied voltages without breaking down, the source junction needs to be carefully designed. A high electric field through the tunnel oxide means that even the electric field at the surface of the silicon is very high, and this can give rise to a leakage current due to band-to-band tunneling (BBT) or breakdown of the source/substrate junction. Source breakdown is one of the major limiting factors to erase time reduction, since the higher the voltage applied to the source, the shorter the erasing time. One of the solutions to the problem is achieved by optimizing the source junction profile to a more gradual one in order to reduce the electric field at the junction.

**Figure 3:**
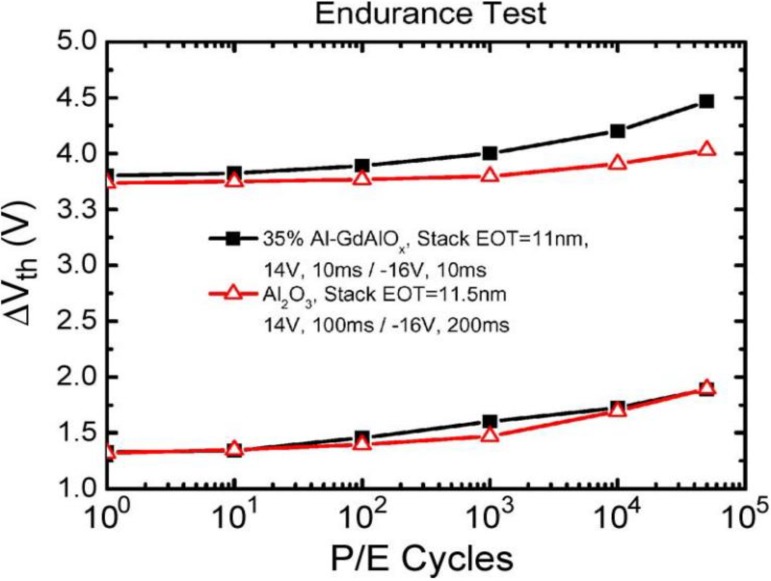
Endurance characteristic of 35% Al-GdAlO*_x_* and Al_2_O_3_ blocking layers. Reused with permission from [35], Copyright 1992, IEEE.

As for the endurance characteristic, the flash memory device is required to maintain its properties on being subjected to repeated program/erase cycles. Figure 3 demonstrates the endurance characteristic of a CTM, although the trend of threshold voltage variation seems not the same as others. When thin dielectrics are repeatedly stressed at high electric fields, oxide, interface, and bulk traps generate in the dielectric. Charge is trapped and released from these traps, and thus it changes the fields across the dielectric. This tends to modify the program/erase characteristics over time, as damage is induced in the dielectric. Flash memories are generally expected to last for 10^5^ cycles without distinct degradation.

Cycling is known to cause a fairly uniform wear-out of the cell performance, mainly due to tunnel oxide degradation, which eventually limits the endurance characteristics [36]. As the experiment was performed applying constant pulses, the variations of program and erase threshold voltage levels are described as “program/erase threshold voltage window closure” and give a measure of the tunnel oxide aging. In particular, the reduction of the programmed threshold with cycling is due to trap generation in the oxide and to interface state generation at the drain side of the channel, which are mechanisms specific to hot-electron degradation. The evolution of the erase threshold voltage reflects the dynamics of net fixed charge in the tunnel oxide as a function of the injected charge. Cycling wear-out can be reduced by proper device engineering and by optimization of the tunnel oxide process. In fact, a high field stress on thin oxide is known to increase the current density at low electric field. The excess current component, which causes a significant deviation from the current-voltage (I-V) curves from the theoretical Fowler-Nordheim characteristics at low field, is known as stress-induced leakage current (SILC). SILC is clearly attributed to stress-induced oxide defects and, as far as a conduction mechanism, it is attributed to a trap assisted tunneling. The main parameters controlling SILC are the stress field, the amount of charge injected during the stress, and the oxide thickness.

**Figure 4:**
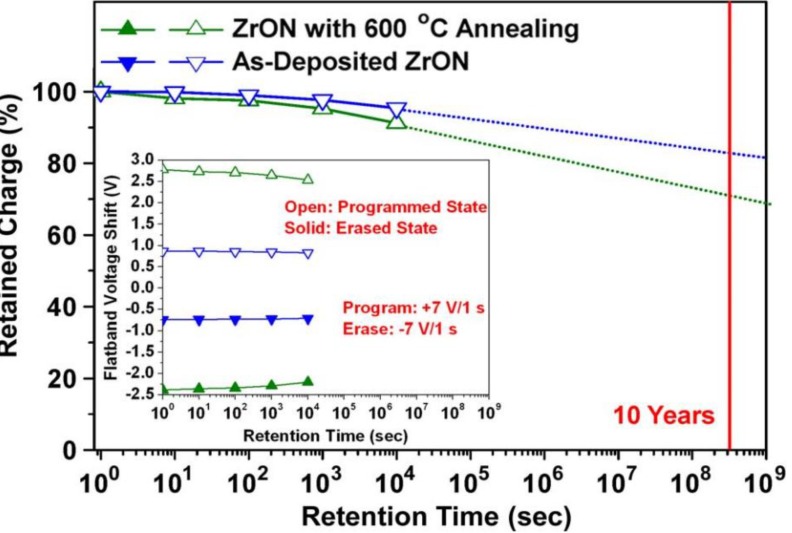
Retention characteristics at 85 °C represented by normalized retained charge for the programmed state of the memory with as-deposited and annealed ZrON films. Reused with permission from [37], Copyright 2010, IEEE.

The retention characteristic is significant in non-volatile memory devices. The ability to retain charge without supplied power is the definition of non-volatile memory. Retention is a metric used to quantify the extent of time expected for which the stored charge is kept in the flash memory. Figure 4 shows an example of retention characteristics of a certain CTM. The charge-loss in the retention mode is determined by tunneling leakage under weak fields through adjacent dielectrics. This charge-loss would be greatly amplified if the dielectrics contained defects or traps, since it would enhance trap-assisted tunneling. A typical retention benchmark for flash memories is calculated for 10 years. Retention tests are further accelerated under higher temperatures. For instance, a 24 h retention measurement at 85 °C is utilized. In updated Flash technology, due to the small cell size, the capacitance is very small and at an operative programmed threshold shift corresponds a number of electrons in the order of 10^3^ to 10^4^. A loss of 20% in this number (around 2–20 electrons lost per month) can lead to a wrong read of the cell and then to a data loss.

Possible causes of charge loss are [36]: (1) defects in the tunnel oxide; (2) defects in the blocking dielectric; (3) mobile ion contamination; and (4) detrapping of charge from insulating layers surrounding the CTL. The generation of defects in the tunnel oxide can be divided into an extrinsic and an intrinsic one. The former is due to defects in the device structure; the latter to the physical mechanisms that are used to program and erase the cell. The best blocking dielectric considering both intrinsic properties and process integration issues has been demonstrated to be a triple layer. The problem of mobile ion contamination has been already solved, taking particular care with the process control, but in particular using high phosphorus content in intermediate dielectric as a guttering element. Electrons can be trapped in the insulating layers surrounding the floating gate during wafer processing, as a result of the so-called plasma damage, or even during the ultra-violet (UV) exposure normally used to bring the cell in a well-defined state at the end of the process. This apparent charge loss disappears if the process ends with a thermal treatment able to remove the trapped charge. Finally, the retention capability of Flash memories has to be checked by using accelerated tests that usually adopt screening electric fields and hostile environments at high temperature.

## 3. Recent Developments

### 3.1. Charge Trapping Layer

Advancements in ultra-thin tunnel oxides during the 1990s have opened the path to improved performance and reliability for non-volatile memory (NVM) based on silicon-oxide-nitride-silicon (SONOS) technology. The storage region for the floating-gate structure is the conducting polysilicon floating-gate electrode, while the SONOS uses a thin silicon-nitride film (Si_3_N_4_) [38]. An advantage of the SONOS device over the floating-gate device is its improved endurance, since a single defect will not cause the discharge of the memory. The stored charge in the SONOS memory device lies in isolated sites within the silicon nitride dielectric. While for the floating-gate device, the case is totally different. A single defect can discharge the stored memory charge of the device due to the conductive properties of the floating polysilicon gate electrode. In the late 1980s and early 1990s, n- and p-channel SONOS devices emerged with write/erase voltages of 5–12 V. Low-voltage SONOS NVMs may be scaled in cell size to 6*F*^2^ (*F* = feature size) and perhaps even smaller.

**Figure 5:**
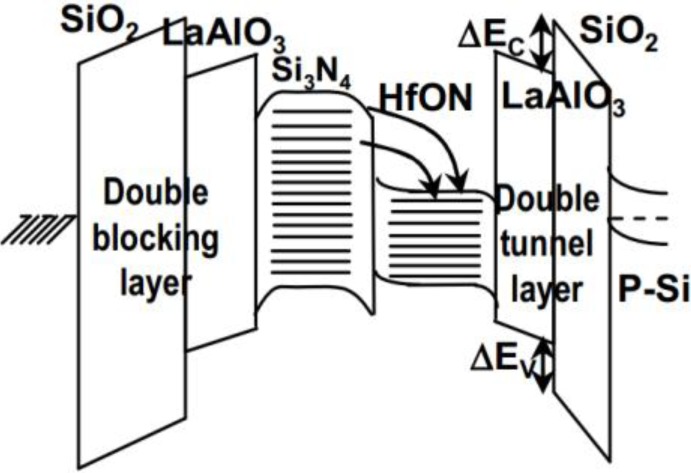
Schematic energy band diagram of double-barrier, double-tunnel and charge-trapping-engineered flash memory. Reused with permission from [39], Copyright 2008, IEDM.

Continuous down-scaling of SONOS devices is required by scaling down the charge-trapping layer to <6 nm, to suppress short-channel effects. This is challenging since the charge trapping deteriorates when the Si_3_N_4_ is made thinner. Very little charge trapping was shown for a 2 nm Si_3_N_4_ layer used as the tunnel layer of SONOS. The high temperature retention also gets worse when the Si_3_N_4_ is thin, due to the higher trap energy in the oxide/Si_3_N_4_/oxide, arising from quantum confinement. A novel charge-trap-engineered flash NVM device was proposed in 2008 [39]. This combines a 5 nm Si_3_N_4_ with a 0.9 nm equivalent oxide thickness (EOT) layer of HfON, within double-barrier and double-tunnel layers, and still shows good retention and a large memory window (schematic energy band diagram of the structure shown in Figure 5). At 150 °C and ±16 V program/erase (P/E), the device showed a P/E speed of 100 μs, an initial Δ*V*_th_ window of 5.6 V and extrapolated 10 year retention of 3.8 V. These results are much better than those of a control charge trap flash device with a single Si_3_N_4_ trapping layer, which had a smaller initial Δ*V*_th_ and poorer 10-year retention.

**Figure 6:**
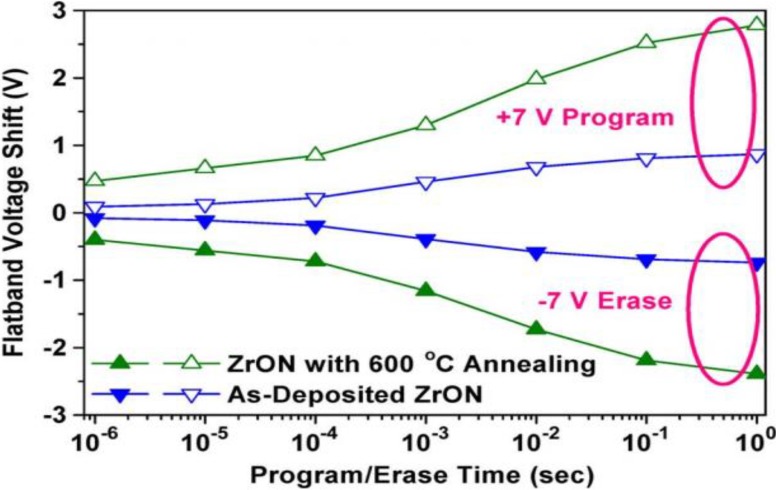
Comparison of P/E transient characteristics for the memory with as-deposited and annealed ZrON films. Reused with permission from [37], Copyright 2010, IEEE.

Si_3_N_4_ has a low dielectric constant (*k* ~ 7), which limits the continual down-scaling of cell size and reduction of operating voltage. To solve these issues, various high*-k* dielectrics with higher *k* value have been widely investigated as charge trapping layer. Other than having a larger conduction-band offset with respect to the tunnel dielectric and, thus, better charge retention, a high*-k* dielectric should also allow a higher electric field over the tunnel dielectric and results in enhanced P/E speed. Most of high*-k* dielectric used in the charge-trapping layer are amorphous phase. However, crystalline high*-k* dielectrics, such as tetragonal and cubic ZrO_2_, have theoretical *k*-values of 46.6 and 36.8, respectively, which are much higher than their amorphous-phase counterpart and will be beneficial to enhance memory performance. A cubic ZrO_2_ film formed by annealing of amorphous ZrON has been investigated as the charge-trapping layer for nonvolatile memory [37]. The memory with a nitrogen-stabilized cubic ZrO_2_ film shows promising performance in terms of 3.81 V hysteresis memory window by ±7 V P/E voltage and 1.98 V flat-band voltage shift by programming at +7 V for 10 ms (P/E transient characteristic shown in Figure 6). Improved performance is mainly due to the greatly enhanced *k*-value of 32.8 and the increased trapping sites provided by grain boundaries.

The charge trapping and tunneling characteristics of HfO_2_ layer that replaces Si_3_N_4_ layer as trap layer were investigated by You *et al.* [40]. The metal-hafnium-oxide-semiconductor (MHOS) structure capacitors with various thicknesses of HfO_2_ layer were fabricated in 2010. The electrical properties of MHOS structure capacitors were compared with those of metal-nitride-oxide-semiconductor (MNOS) structure capacitors. The gate leakage current of MHOS capacitor due to tunneling was significantly reduced by stacking the HfO_2_ trap layer on thin SiO_2_ tunnel layer. The MHOS capacitors showed a larger memory window than the MNOS capacitors at the same trap layer thickness, because the HfO_2_ layer has better charge trapping efficiency than the Si_3_N_4_ layer. Especially, the Si_3_N_4_ trap layer is difficult to reduce the film thickness below 4 nm thick due to enormous increase of the tunneling current. However, the HfO_2_ trap layer can reduce the thickness below 4nm. It is also observed that the ultrathin HfO_2_ trap layer with a thickness of 2 nm stored almost the same charges with Si_3_N_4_ layer with a thickness of 7 nm. Even in ultrathin trap layer with a thickness of 2 nm, the memory window of HfO_2_ layer (0.67 V) is much higher than that of the Si_3_N_4_ layer (0.025 V) and shows a similar value to 7 nm thick Si_3_N_4_ trap layer.

**Figure 7:**
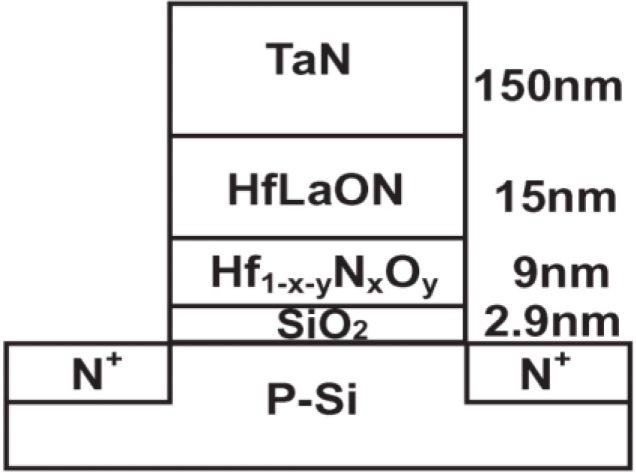
.TaN/HfLaON/Hf_0.35_N_0.10_O_0.55_/SiO_2_/Si MONOS transistors device structure. Reused with permission from [41], Copyright 2008, IEEE.

Yang *et al.* [41] have studied the nitrogen composition dependence of the characteristics of TaN/HfLaON/Hf_1*−x−y*_N*_x_*O*_y_*/SiO_2_/Si (MONOS) memory devices in 2008. To overcome performance degradation of increased P/E voltage and write speed, the use of an Al(Ga)N storage layer was proposed which has deeper ∆*E*_C_ than Si_3_N_4_. This improved the P/E voltage and write speeds in such deep-trap MONOS devices. Unfortunately, further improvement beyond Al(Ga)N is limited since most of the metal–nitrides are metallic. To avert this problem, a higher *k* Hf_1−*x*−*y*_N*_x_*O*_y_* dielectric was used for MONOS applications, where even lower P/E voltages and better high temperature retention can be achieved. Here, the nitride composition in the Hf_1*−x−y*_N*_x_*O*_y_* beyond was varied to investigate how this alters the characteristics. Transistor device structure is shown in Figure 7. Increasing the N composition in the Hf_1*−x−y*_N*_x_*O*_y_* trapping layer has improved both the memory window and high-temperature retention. The Hf_0.3_N_0.2_O_0.5_ MONOS device showed ±9 V P/E voltage, 100 μs P/E speed, large initial 2.8 V memory window, and a ten-year expected retention of 1.8 V at 85 °C or 1.5 V at 125 °C. Good endurance was obtained, as is evident from the still memory windows of 2.4 and 1.7 V after 10^5^ cycles at ±9 V 100 μs P/E for Hf_0.3_N_0.2_O_0.5_ and Hf_0.35_N_0.10_O_0.55_ MONOS devices.

The polysilicon–oxide–high*-k*–oxide–silicon (SOHOS) structure, with hafnium oxide (HfO_2_) as the charge-storage layer, demonstrates a superior charge-storage capability at low voltages, faster programming, and less over-erase problems compared to the conventional SONOS devices. However, the SOHOS device has a poorer charge-retention capability than the SONOS one. While using aluminum oxide (Al_2_O_3_) as the charge-storage layer results in a SOHOS structure with an improved charge-retention performance, but at the expense of a slower programming speed. Therefore, by adding a small amount of aluminum to HfO_2_ to form hafnium aluminum oxide (HfAlO), the SOHOS structure with HfAlO as the charge-storage layer can combine the advantages of both HfO_2_ and Al_2_O_3_, like fast programming speed and good charge-retention capability [42]. From the programming (*V*_g_ − *V*_fb_ = 6 V) and erasing (*V*_g_ − *V*_fb_ = −6 V) characteristics, respectively, HfAlO as charge-trapping layer devices show the fastest programming and erase speed while the Al_2_O_3_ device is the slowest. The memory window is 3 V at 100 ms of P/E process. The write/erase (W/E) endurance characteristic of the HfAlO device shows no discernible difference from that of the Si_3_N_4_ device. Both Si_3_N_4_ and HfAlO devices show a negligible degradation in the threshold voltage window after 10^4^ W/E cycles. The dominant charge-storage mechanism is due to electron trapping in the bulk of the HfAlO layer, rather than negative charge trapping at the tunnel oxide/high-*k* interface, which will be independent of the HfAlO thickness. The charge-retention performance of the SOHOS device also degrades with the decreasing HfAlO thickness. This can be understood from the fact that, for SOHOS devices with a thicker HfAlO layer, electrons that are trapped within the bulk may have to tunnel through a longer distance through the HfAlO layer to the tunnel SiO_2_ and the silicon substrate. The initial *V*_fb_ after programming is 3.5 V for 12.5 nm HfAlO and after 10^4^ s the value retains at 3.4 V.

**Figure 8:**
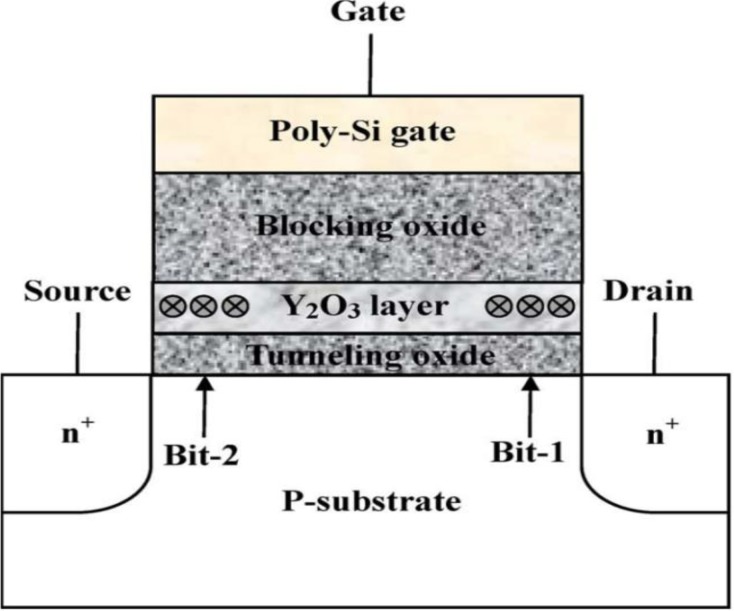
Schematic representation of flash memory cell structure using the Y_2_O_3_ as a charge-trapping layer. Reused with permission from [43], Copyright 2008, IEEE.

The rare earth oxides such as yttrium oxide (Y_2_O_3_) are attractive candidates for the trapping layer memory based on thermodynamic stability consideration, a high dielectric constant of 18, a high conduction band offset over 2 eV, and a low lattice mismatch with silicon. Pan *et al.*, proposed a novel high*-k* Y_2_O_3_ SONOS-type flash memory in 2008 [43]. Schematic representation of flash memory cell structure is shown in Figure 8. These high*-k* Y_2_O_3_ SONOS-type memories exhibited large threshold voltage shifting, almost negligible read and gate disturb, excellent data retention (charge loss of ~4% measured time up to 10^4^ s and at room temperature, expected ~22% charge loss for ten years at 125 °C), and superior endurance characteristics (program/erase cycles up to 10^5^) because of the higher probability for trapping charge carriers. For the condition of *V*_D_ = 6 V and *V*_G_ = 8 V at 1 ms, it is obvious that a high-k Y_2_O_3_ SONOS-type memory after N_2_ annealing exhibits a larger memory window of 2.43 V compared with other annealing gases. This is due to more electrons trapped in the Y_2_O_3_ layer. Moreover, excellent erase speed of approximately 1ms can be obtained for Y-silicate trapping storage layer memory prepared at a N_2_ ambient annealing and operated at *V*_D_ = 8 V and *V*_G_ = −3 V. The values of *V*_th_ in the program and erase states for Y-silicate charge trapping layer memory prepared at a N_2_ gas annealing did not increase significantly up to 10^5^ P/E cycles. The trapped electrons from the Y-silicate layer are almost removed during the erase process. In contrast, the memory window underwent a narrowing after 10^5^ cyclic operations for high-*k* Y_2_O_3_ SONOS-type memory after O_2_ annealing. An yttrium silicate trapping storage layer memory annealed in N_2_ gas exhibits a small charge loss of about 4% measured time up to 10^4^ s and at 25 °C. This result is attributed to the combined effects of the tight embrace of the Y_2_O_3_ film by the sufficiently deep trap energy level. Although the thickness of tunnel oxide is 2 nm, almost no significant lateral or vertical charge migration occurs. At the temperature of 125 °C, SONOS-type memory prepared under an Y_2_O_3_ trapping layer and annealed in N_2_ gas has a lower charge loss of 22% during the program state compared with other annealing gases. This indicates that the yttrium silicate charge-trapping layer can tightly catch the tunneling electrons. Therefore, the trapped electrons by the high-*k* Y_2_O_3_ SONOS-type memory devices cannot easily escape, and the exhibited charge loss percentage is low.

One difficulty in SONOS is the small conduction band discontinuity at the Si_3_N_4_/SiO_2_ interface, which causes the charge leak out from the shallow trap levels of Si_3_N_4_. To overcome this problem, high-*k* dielectric materials, such as ZrO_2_, HfO_2_, and Y_2_O_3_ are promising candidates to replace a Si_3_N_4_ film as the charge trapping layer of SONOS Flash devices. Such high-*k* dielectric films can achieve improved charge trapping characteristics than the Si_3_N_4_ films because of their sufficient densities of trap states and deep trap energy levels, giving rise to better data retention. In recent years, rare-earth oxides have attracted much interest in research for complementary metal oxide semiconductor applications of high-*k* materials due to their large energy band gaps and high dielectric constants. Among them, Tb_2_O_3_ possesses desirable properties for charge trap Flash device application, such as a relatively high dielectric constant, a large band gap, a large conduction band offset with regard to silicon and good thermal stability with Si. A metal-oxide-high-k-oxide-silicon (MOHOS)-type memory structure fabricating a high-*k* Tb_2_O_3_ charge-trapping layer for flash memory applications was reported afterwards [44]. The high-*k* Tb_2_O_3_ MOHOS-type memories annealed at 800 °C exhibited large threshold voltage shifting (memory window of 1.41 V operated at *V*_g_ = 8 V at 0.1 s), excellent data retention (charge loss of 10% measured time up to 10^4^ s and at 85 °C), and good endurance characteristics (program/erase cycles up to 10^5^) because of the high probability and deep trap level for trapping the charge carrier due to the formation of the crystallized Tb_2_O_3_ with a high dielectric constant of 11.8.

**Figure 9:**
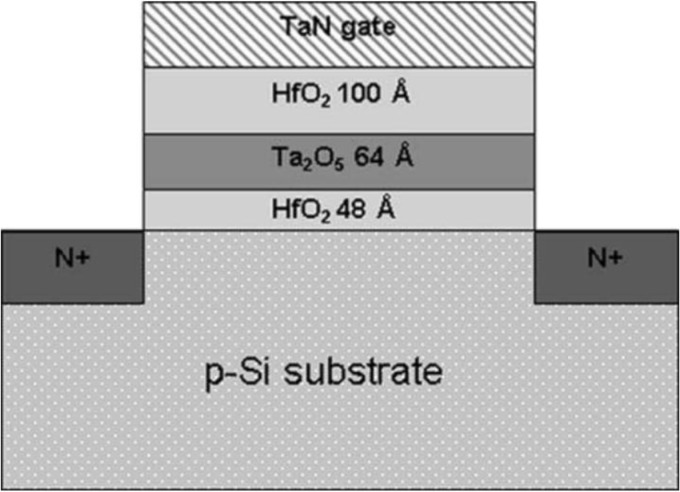
Fabricated metal-HfO_2_-Ta_2_O_5_-HfO_2_-Si nMOS transistor structure. Reused with permission from [45], Copyright 2004, IEEE.

Several efforts have been made to improve both the retention time and the programming speed in SONOS devices. However, few of these solutions deal with the bottom tunneling oxide layer, which has already been scaled down to below 2 nm. Further scaling tunneling oxide thickness and at the same time meeting the ten-year retention is a challenge even with these approaches. A novel memory structure, based on the band engineering considerations of HfO_2_ for the tunneling and blocking layer as well as Ta_2_O_5_ for the charge storage layer, was proposed to address these problems in 2004 [45]. Wang *et al.*, demonstrate for the first time that HfO_2_ with its low charge barrier height and Ta_2_O_5_ with its deep-trap energy level and proper band offset alignments, are good candidates to replace the traditional SiO_2_-Si_3_N_4_-SiO_2_ (ONO) stack to achieve faster programming and better retention performance simultaneously. The transistor structure is shown in Figure 9. The fabricated devices can be programmed as fast as 1 ns and erased from 10 ns at an 8 V gate bias. The retention decay rate of this device is improved by a factor more than three as compared to the conventional SONOS type devices.

The nanocrystals embedded dielectric structure has been proposed to replace the poly-Si floating-gate structure. Conventional high-*k* materials, such as ZrO_2_ and HfO_2_, have low crystallization temperatures, e.g., lower than 600 °C, which is a reliability concern. Various nanocrystalline semiconductive and conductive materials, such as Ru, RuO, indium tin oxide, and ZnO, have been embedded into high-*k* films as the electron-or hole-trapping media. Since molybdenum oxide (MoO*_x_*) has a high work function, it can be a good charge trapping medium in the high-*k* film: ZrHfO [46]. The nc-MoO*_x_* sample shows a large *V*_FB_ shift of 0.52 V after the 8 V stress, which corresponds to the hole trapping density of 1.14 × 10^12^ cm^−2^. On the other hand, for the same sample, a very small positive *V*_FB_ shift, *i.e.*, 0.04 V is observed after the +8 V stress. About 54% of trapped holes (under the 8 V stress condition) remain in the sample after 10 years. In addition, about 52% of trapped holes (under the 7 V stress condition) remain in the sample after 10 years.

Extensive researches have been performed to study high-*k* dielectrics instead of Si_3_N_4_ as CTL for further scaling down of the memory dimensions and improving its charge-trapping efficiency, e.g., Y_2_O_3_ (*k* ~ 18), HfON (*k* ~ 22), Pr_2_O_3_ (*k* ~ 15), Nd_2_O_3_ (*k* ~ 16), Er_2_O_3_ (*k* ~ 13), ZrO_2_ (*k* ~ 37), and Ta_2_O_5_ (*k* ~ 22). Unfortunately, few dielectrics proposed as CTL show a dielectric constant beyond 30. Among various high-*k* dielectrics, SrTiO_3_ is well-known for its high dielectric constant (*k* ~ 140) and zero band-offset with respect to silicon, both of which are desirable as CTL for memories to improve the program/erase (P/E) speeds and retention property. Nitrogen incorporation also plays an important role in the characteristics of SrTiO_3_ because it may induce more traps in the band gap through substitution of oxygen by nitrogen. Nitrided SrTiO_3_ [47] showed a larger memory window (8.4 V at ±10 V sweeping voltage), higher P/E speeds (1.8 V at 1 ms +8 V) and better retention properties (charge loss of 38% after 10^4^ s), due to the nitrided SrTiO_3_ thin film exhibiting higher dielectric constant, higher deep-level traps induced by nitrogen incorporation, and suppressed formation of Ti silicate between the CTL and SiO_2_ by nitrogen passivation.

BaTiO_3_ is well known for a high dielectric constant (*k* > 100) with strong scaling ability and its negative band offset with respect to Si, thus, leading to a large barrier height relative to SiO_2_. In addition, Zr-doped BaTiO_3_ has also attracted increasing interest because the isovalent substitution of Ti with Zr in BaTiO3 can shift the Curie temperature below room temperature, thus, making the dielectric paraelectric at room temperature without fatigue problems [48]. Also, Zr-doped BaTiO_3_ has been demonstrated to have a lower leakage current than BaTiO_3_ while maintaining a comparable dielectric constant. Compared with the device without Zr incorporation, the one with Zr incorporation showed a similar memory window (8.3 V at ±12 V for 1 s), but higher program speed at low gate voltage (3.2 V at 100 μs + 6 V) and better endurance and data retention (charge loss of 6.4% at 150 °C for 10^4^ s), due to the Zr-doped BaTiO_3_ exhibiting higher charge-trapping efficiency and higher density of traps. Under a ±12 V 100 μs stress pulse the P/E windows of the Zr-BTO sample before and after the 10^5^-cycle stressing are 6.4 and 6.6 V, respectively, and negligible degradation happens.

**Figure 10:**
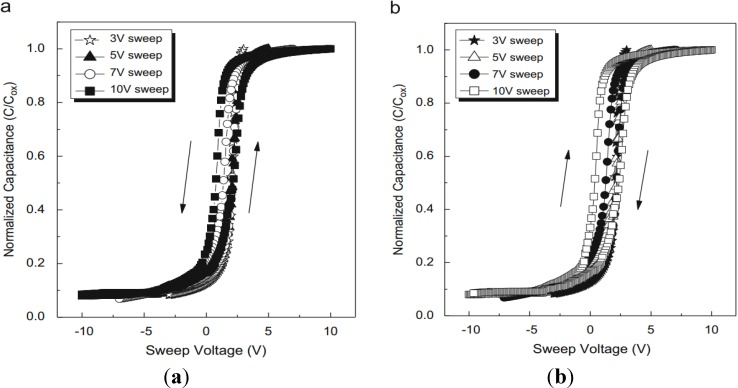
The C-V hysteresis of: (**a**) 5 nm and (**b**) 15 nm-thick Al_2_O_3_ blocking oxide on the 5 nm La_2_O_3_ trap layer/5 nm Al_2_O_3_ tunnel oxide structure as a function of the double-sweep voltage. Reused with permission from [49], Copyright 2010, Materials Science in Semiconductor Processing.

Using the high*-k* dielectric as a trap layer has advantages of decreased program/erase voltage and improved retention characteristics as compared to the SONOS devices. These advantages can lead to the superior charge trap characteristics. However, the ONO structure must have over three steps to make three layers (bottom SiO_2_-thermal oxidation, Si_3_N_4_-LPCVD, and top SiO_2_-PECVD). On the other hand, multi-stacked films using the high-*k* materials are generally able to make a one-step approach due to the oxide base. This means that a three-gas line (two sources line + one oxygen line) can make three layers. In order to investigate charge trap characteristics with various thicknesses of blocking and tunnel oxides for application to nonvolatile memory devices, Kim *et al.*, fabricated two multi-stack films, respectively [49]. One is Al_2_O_3_(5 and 15 nm)/La_2_O_3_(5 nm)/Al_2_O_3_(5 nm). The other is Al_2_O_3_(15 nm)/La_2_O_3_(5 nm)/Al_2_O_3_(5, 7.5 and 10 nm). The optimized structure was 15 nm Al_2_O_3_ blocking oxide/5 nm La_2_O_3_ trap layer/5 nm Al_2_O_3_ tunnel oxide films. The maximum memory window of this film of about 1.12 V was observed at 11 V for 10 ms in the program mode and at 13 V for 100 ms in the erase mode (the C-V hysteresis as a function of double-sweep voltage shown in Figure 10). The operation conditions of the P/E cycles were 11 V for 10 ms in the program mode and 13 V for 100 ms in the erase mode. The difference of the threshold voltages between the program and erase mode was about 1.2 V and was maintained over 10^4^ P/E cycles.

The programming characteristics of CTM devices can be enhanced by the high-*k* CT layer due to its larger trap density and smaller band offset to Si. However, the retention characteristic is still an issue because high-*k* material suffers lower crystalline temperature and shallower defect level. Therefore, a Si_3_N_4_/high-*k* material stacked CT layer was proposed to improve the retention characteristics since Si_3_N_4_ has deeper trap level and higher crystalline temperature and provides an effective barrier for high-*k* material such as HfO_2_. Moreover, a faster erase speed can also be achieved by its smaller valence band offset for Si_3_N_4_ to Si. Multilevel cell characteristics can be obtained by inserting Al_2_O_3_ into Si_3_N_4_ (*i.e.*, Si_3_N_4_/Al_2_O_3_/Si_3_N_4_ trapping layer) due to the modulated trapping charge distribution. CT NVM devices with Si_3_N_4_/Al_2_O_3_/high-*k* CT layer is then proposed to further scale down [50]. The sample with Si_3_N_4_/Al_2_O_3_/HfO_2_ CT layer has the fastest programming speed since it can modulate the trapped charge distribution. By inserting an Al_2_O_3_ layer between Si_3_N_4_ and HfO_2_, most of the injected electrons are trapped at the Si_3_N_4_/Al_2_O_3_ interface and thus lower the leakage current. The sample performs best because there is an additional barrier provided by Al_2_O_3_ to suppress the detrapping of electrons in HfO_2_.

In conclusion, several groups’ research output in the paper and the detailed specification and current status have been listed in a Table 3 (include high-*k* as charge trapping layer, deposition technique, program/erase, endurance and retention characteristics). 

**Table 3:** Current research status of high-*k* dielectrics as charge-trapping layer [37,38,39,40,41,42,43,44,45,46,47,48,49,50].

High-*k* as CT Layer	Deposition Technique	Program/Erase Characteristic	Endurance Characteristics	Retention Characteristics
Si_3_N_4_ + HfON	The layers of the TaN-[SiO_2_-LaAlO_3_]-[Si_3_N_4_-HfON]-[LaAlO_3_-SiO_2_]-Si devices comprised 2.5 nm of thermal SiO_2_, 2.5 nm of PVD LaAlO_3_, 5 nm of reactive PVD HfON_0.2_ and 5 nm of Si_3_N_4_ by LPCVD. Then 8 nm LaAlO_3_ by PVD, 5 nm SiO_2_ by PECVD, and 200 nm TaN by PVD. This was followed by standard gate definition, self-aligned P+ implantation and an RTA.	150 °C and ± 16 V program/erase (P/E), the device showed P/E speed of 100 μs, an initial Δ*V*_th_ window of 5.6 V.	a large 10^5^-cycle window of 4.9 V and 10^3^-cycled 10-year retention window of 4.1 V, at ±16 V 100 μs P/E.	An initial Δ*V*_th_ window of 5.6 V is set and later extrapolated 10 year retention of 3.8 V.
ZrON	ZrON film was deposited in a reactive magnetron sputtering system as the charge-trapping layer. To form the ZrON film, a pure Zr target was sputtered in an oxygen (4-sccm)/nitrogen (12-sccm)/argon mixture (24-sccm) gas ambient. A subsequent annealing was performed on some as-deposited samples in a N_2_ ambient at 600 °C for 30 min.	3.81 V hysteresis memory window by ±7 V P/E voltage and 1.98 V flat-band voltage shift by programming at +7 V for 10 ms.	negligible degradation of the memory window up to 10^5^ P/E cycles from the endurance measurement, in which ±7 V with 10 ms gate pulse-width used.	28.6% charge loss after ten-year operation at 85 °C.
HfO_2_	After a growth of thermal oxide with a thickness of 3 nm at 700 °C in dry O_2_ ambient, a deposition of HfO_2_ layers ranging from 8 to 2 nm was followed by atomic layered chemical vapor deposition method at 300 °C to evaluate the effect of thickness on the charge trapping and tunneling characteristics.	The memory window for 8 nm HfO_2_ layer is 1.5 V at high frequency of 1 MHz and sweep voltage of ±5 V.	insufficient data given.	An initial memory window of 6.16 V; after 10 years, the memory window of 4.26 V. Charge loss rate of 30.8%.
Hf_1−*x*−*y*_N*_x_*O*_y_*	A 9-nm Hf_1−*x*−*y*_N*_x_*O*_y_* layer was deposited by reactive sputtering under a mixed O_2_ and N_2_ conditions with different O_2_/N_2_ ratios to study the N% dependence of the MONOS device integrity.	The Hf_0.3_N_0.2_O_0.5_ MONOS device showed ±9 V P/E voltage, 100 μs P/E speed, large initial 2.8 V memory window.	memory windows of 2.4 V and 1.7 V after 10^5^ cycles at ±9 V 100 μs P/E for Hf_0.3_N_0.2_O_0.5_ devices and Hf_0.35_N_0.10_O_0.55_ MONOS devices, respectively.	a ten-year expected retention of 1.8 V at 85 °C or 1.5 V at 125 °C.
HfAlO_2_	For the SOHOS device, pure HfO_2_ and Al_2_O_3_ films were deposited by the ALD, while HfAlO films were deposited by metal organic chemical vapor deposition using a single cocktail source. The Al_2_O_3_ concentration in HfAlO was kept at 10%.	From the programming (*V*_g_ − *V*_fb_ = 6 V) and erasing (*V*_g_ − *V*_fb_ = −6 V), memory window is 3 V at 100 ms of P/E process.	HfAlO devices show a negligible degradation in the threshold voltage window after 10^4^ W/E cycles.	The initial *V*_fb_ after programming is 3.5 V for 12.5 nm HfAlO and after 10^4^ s the value retains at 3.4 V.
Y_2_O_3_	A 3 nm Y_2_O_3_ film was deposited on the tunneling oxide by sputtering with yttrium target in a system with a base pressure of 9.8 × 10^−3^ m·bar at room temperature. The sputtering process was performed in diluted O_2_(Ar/O_2_ = 25/5) ambient sputtering power of 100 W, at sputter rates of about 2.5 Å/min. Rapid thermal process anneal of 700 °C in N_2_,O_2_, or N_2_O ambient for 30 s was then performed to transform Y_2_O_3_ film into Y-silicate charge trapping layer.	For the condition of *V*_D_ = 6 V and *V*_G_ = 8 V at 1ms, a high-*k* Y_2_O_3_ SONOS-type memory after N_2_ annealing exhibits a larger memory window of 2.43 V compared with other annealing gases.	The values of *V*_th_ in the program and erase states for Y-silicate charge trapping layer memory prepared at a N_2_ gas annealing did not increase significantly up to 10^5^ P/E cycles.	An yttrium silicate trapping storage layer memory annealed in N_2_ gas exhibits a small charge loss of about 4% measured time up to 10^4^ s and at 25 °C. At the temperature at 125 °C, SONOS-type memory prepared under an Y_2_O_3_ trapping layer and annealed in N_2_ gas has a lower charge loss of 22% during the program state compared with other annealing gases.
Tb_2_O_3_	The 8 nm Tb_2_O_3_ film was deposited on the tunneling oxide by reactive sputtering in diluted O_2_ from a terbium target as a charge trapping layer. The wafers were annealed in O_2_ gas for 30 s at 800 °C by rapid thermal annealing.	Threshold voltage shifting (memory window of 1.41 V operated at *V*_g_ = 8 V at 0.1 s).	The values of *V*_FB_ in the program and erase states did not increase significantly up to 10^5^ P/E cycles.	Charge loss 10% measured time up to 10^4^ s at 85 °C.
Ta_2_O_5_	After standard clean, the substrate was first annealed in NH_3_ at 700 °C, 10s to improve interface quality, followed by CVD HfO_2_ tunneling oxide layer formation using Hafnium t-butoxide (Hf(OC_4_H_9_)_4_ at 500 °C. The Ta_2_O_5_ charge-trapping layer was formed by oxidation of physical vapor deposition (PVD) Ta at 550 °C.	Memory windows of about 0.8V when the device is programmed with ±8 V-1-ms gate pulse; The device can be written starting from 10 us and erased from 1 ms for 6 and 7 V.	Memory window has no obvious shrink after 10 write/erase cycles with 8 V 1 ms stress at room temperature.	Retention characteristics at room temperature and 85 °C demonstrate a decay rate of 50 mV/dec. Memory window extrapolated at ten years is 0.64 V (initial 0.8 V) at room temperature, and 0.42 V at 85 °C.
MoO*_x_*	The embedded MoO*_x_* layer was sputtered from the Mo target in Ar/O_2_(1:1) at 5 mTorr and 100 W for 15 s. The as-deposited MoO*_x_* layer was transformed into nanocrystalline MoO_3_, after the PDA step at 800 °C for 1 min in the pure N_2_ ambient by rapid thermal annealing.	The nc-MoO*_x_* sample shows a large *V*_FB_ shift of −0.52 V after the −8 V stress, which corresponds to the hole trapping density of 1.14 × 10^12^ cm^−2^. On the other hand, for the same sample, a very small positive *V*_FB_ shift, *i.e.*, 0.04 V is observed after the +8 V stress.	insufficient data given.	About 54% of trapped holes (under the −8 V stress condition) remain in the sample after 10 years. Also, about 52% of trapped holes (under the −7 V stress condition) remain in the sample after 10 years.	
SrTiO_3_	3 nm SrTiO_3_ was deposited on the SiO_2_ by reactive sputtering using a SrTiO_3_ target in a mixed Ar/N_2_ (4/1) or Ar/O_2_ (4/1) ambient.	memory window (8.4 V at ±10 V sweeping voltage), P/E speeds (1.8 V at 1 ms +8 V).	memory window after 10^5^ cycles is 2.13 V compared with initial value of 2.07 V.	charge loss of 38% after 10^4^ s.	
BaTiO_3_	10 nm Zr-doped BaTiO_3_ was deposited on the SiO_2_ by reactive sputtering using BaTiO_3_ and Zr targets in a mixed Ar/O_2_ ambient, and the atomic ratio of Zr and Ti was determined to be 1/3. The sample went through PDA in a N_2_ ambient at 900 °C for 30 s.	memory window (8.3 V at ±12 V for 1 s), but higher program speed at low gate voltage (3.2 V at 100 μs +6 V).	Under a ±12 V 100 μs stress pulse the P/E windows of the Zr-BTO sample before and after the 10^5^-cycle stressing are 6.4 and 6.6 V, respectively, and negligible degradation happens.	charge loss of 6.4% at 150 °C for 10^4^ s.	
Al_2_O_3_ + La_2_O_3_ + Al_2_O_3_	Al_2_O_3_/La_2_O_3_/Al_2_O_3_ films were deposited on (100) ntype Si wafers (SILTRON, Korea) by a MOCVD system. N_2_ was used as a carrier gas for La and Al precursor.	maximum memory window of this film of about 1.12 V was observed at 11 V for 10 ms in the program mode and at −13 V for 100 ms in the erase mode.	memory window after 10^4^ cycles is 1.2 V compared with initial value of 1.12 V.	insufficient data given.	
Si_3_N_4_ + Al_2_O_3_ + HfO_2_	Si_3_N_4_ was deposited by low-pressure chemical vapor deposition, and Al_2_O_3_ and HfO_2_ were deposited using the metal–organic chemical vapor deposition method. High-temperature annealing was performed on all samples in a N_2_ ambient for 30 s at 900 °C by rapid thermal anneal.	at *V*_P_ = 16 V and *V*_E_ = −16 V for 1 s, memory window of this film of about 10 V, P/E speeds (6.1 V at 1 ms +8 V).	no sufficient data given.	charge loss of 4% after 10^4^ s.	

### 3.2. Blocking Layer

SiO_2_ is the first dielectric as blocking layer in the flash memory. However, the large tunneling current through SiO_2_ is not acceptable upon continually scaling down of the dimensions of the flash memory. Since the erase speed for this type of SONOS devices is determined by the competition of the direct band-to-band tunneling current through a tunnel oxide and the unwanted Fowler-Nordheim tunneling current through a blocking oxide, the tunnel oxide thickness is fundamentally required to be less than 2 nm for a stable erase operation. Therefore, numerous attempts have been tried to replace thermal oxides by new dielectrics or related new structures.

Among the group III candidate dielectrics, such as alumina (Al_2_O_3_), are very stable and robust materials, and have been extensively studied for many applications. Regarding its usefulness as an alternate gate dielectric, Al_2_O_3_ has many favorable properties, including a high band gap, thermodynamic stability on Si up to high temperatures, higher dielectric constant (*k* ~ 9) than SiO_2_ (*k* ~ 3.9), and is amorphous under the conditions of interest. In 2005, Lee proposed a SiO_2_/SiN/Al_2_O_3_ (SANOS) device structure that can make fast P/E operation by FN tunneling mechanism possible even at a thicker tunnel oxide over 3 nm [51]. This is achieved by employing a high*-k* dielectric material, especially Al_2_O_3_ replacing the top silicon oxide for a blocking layer. Electron injection is suppressed effectively by the Al_2_O_3_ and the leakage current characteristics of the Al_2_O_3_ layer are sufficient to block the electron injection from the gate in the reasonable erase voltage range. Furthermore, this structure achieves longer data retention and realizes lower voltage programming than the conventional SiO_2_/SiN/SiO_2_ structure. Excellent retention characteristics could be achieved by using a thicker (>2 nm) tunnel oxide and thus, suppressing the electron discharging due to the band-to-band direct tunneling mechanism.

High*-k* dielectrics as the blocking oxide layer proportionally reduced the electric field across the blocking oxide with its dielectric constant. Therefore, electron injection from the gate during erase can be effectively suppressed, which will generally in turn enhance the erase speed. In addition, using a physical thicker blocking layer film minimizes the electron leaking out to the gate during retention. Faster operation speed has been observed by replacing the SiO_2_ blocking layer with Al_2_O_3_. However, the drawback is that Al_2_O_3_ only has *k* ~ 8–10, and would therefore make it a relatively short-term solution for industry’s needs ~1–2 generations. If no longer-term solution is available by the time that a replacement is required, however, such a short-term solution may indeed be suitable. Among all the high*-k* dielectric candidates, rare earth oxides are recently of research interests as the gate dielectrics in CMOS applications, owing to their high*-k* dielectric constant and large energy band gap. Gd_2_O_3_ has advantages as the blocking layer of the SONOS devices. One novel technique to engineer the Gd_2_O_3_ film was introduced by Pu *et al.*, in 2009, for further improvement of the device performance [52]. Aluminum-doped Gd_2_O_3_ is an attractive dielectric material as the blocking oxide of SONOS-type Flash memory, showing a superior charge retention property, as well as the improved operation speed. The blocking oxide layer is 22%, 35%, and 75% GdAlO*_x_* films (the entire stack EOTs are 10.8, 10.5, and 13 nm, respectively). The physical thicknesses of the 22%, 35%, and 75% GdAlO*_x_* layers are 14.9, 11.1, and 14 nm, and their calculated *k* values are 17, 14, and 10, respectively. After gate stack patterning and source/drain implantation, dopant activation anneal was done at 850 °C for 30 min in a N_2_ ambient in a furnace tube. Forming gas (10% H_2_ in N_2_) annealing at 420 °C for 10 min was added before wafer fab out. With a proper optimization of the structure, it has been shown that the GdAlO*_x_* blocking layer can be a good candidate material for the further scaled charge-trap-type Flash memory devices.

Hafnium silicon oxide (*k* ~ 12) has been evaluated as the blocking oxide recently. However, the devices exhibited a poor retention performance. Lanthanum-based dielectrics (LaAlO*_x_*) are chosen as the blocking oxide due to their relatively large conduction band offset and high dielectric constant [53]. Compared to Al_2_O_3_ blocking oxide, LaAlO*_x_* not only exhibits faster program speed, wider *V*_th_ window, and more robustness to voltage stress but also has a better retention performance when the temperature is below 120 °C, particularly at 85 °C. It is also found that the retention property is critically determined by the conduction band offset of a blocking oxide, which is caused by the shallow trapping energy depth inside the nitride calculated to be 0.6–0.75 eV below the conduction band edge. LaAlO*_x_* blocking oxides were deposited using an atomic layer deposition method. During the ALD process, La(iPrCp)_3_, Al(CH_3_)_3_, and H_2_O were used as lanthanum precursor, aluminum precursor, and oxidant, respectively. After gate stack patterning, S/D implantation with arsenic, dopant activation annealing in a furnace (850 °C for 30 min in a N_2_ ambient), and forming gas anneal was performed at 420 °C for 30 min in a N_2_/H_2_ ambient. The physical thickness of LaAlO were 17 nm, and they exhibit the same gate stack equivalent oxide thickness (EOT_total_ = 12.2 nm). The atomic ratio of La/(La + Al) in a LaAlO*_x_* film was around 55%.

Among rare-earth metal oxides, yttrium oxide (Y_2_O_3_) shows the strongest affinity for oxygen, no humidity absorption (compared with La_2_O_3_), and no magnetic characteristics (compared with Gd_2_O_3_), thus attracting increasing interest as a blocking layer. The main shortcomings of Y_2_O_3_ lie in its poor thermal property and relatively small band-gap (~6 eV) compared with Al_2_O_3_. A novel method to engineer Y-doped Al_2_O_3_ film as the blocking layer was proposed by Huang *et al.*, in 2011, via co-sputtering Y and Al targets in an Ar/O_2_ mixed ambient for further improvement of the dielectric performance [54]. Compared with Al_2_O_3_ and Y_2_O_3_ films, the optimized Y*_x_*Al*_y_*O film shows lower interface-state density, lower bulk charge-trapping density, higher dielectric constant, and smaller gate leakage, due to the suppressed interlayer and good thermal property ascribed to appropriate Y and Al contents in the thin film. Y*_x_*Al*_y_*O with different Y contents was deposited on the wafers by co-sputtering of Al and Y targets in a mixed ambient (Ar/O_2_ = 24:10). To explore the ability of Y doping to consume the interlayer, a high ratio of oxygen was used for the reactive sputtering because it could lead to a thicker interlayer. Al sputtering power was fixed at 25 W, while Y sputtering power was set as 20, 25, and 30 W to obtain samples with different amounts of Y incorporation. Following that, the samples received post-deposition annealing (PDA) at 950 °C for 30 s in N_2_ ambient. All the samples had similar EOT (~3.1 nm).

### 3.3. Tunneling Layer

**Figure 11:**
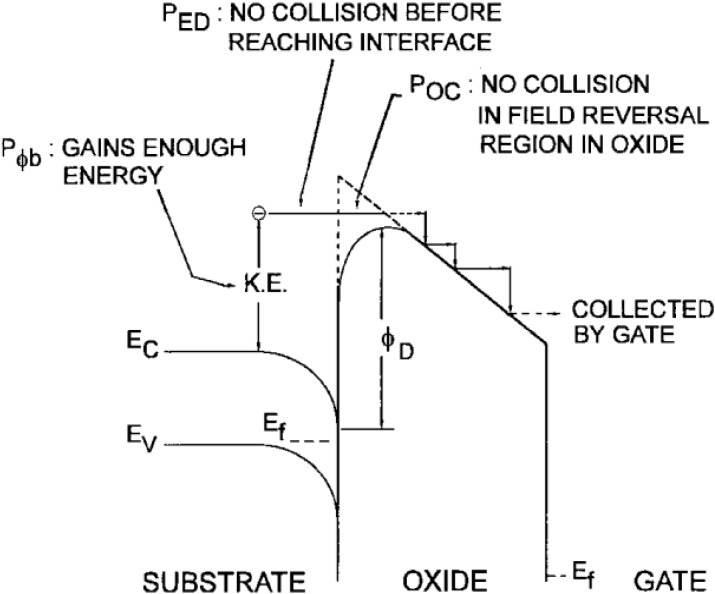
A schematic energy band diagram describing the three processes involved in electron injection. Reused with permission from [34], Copyright 1997, IEEE.

There are two dominant charge-injection mechanisms available to change the charge content of the charge trapping memory: channel hot electron (CHE) and Fowler-Nordheim (F-N) tunneling. The fundamental of CHE is to provide enough energy to the channel electrons to overcome the tunnel oxide (silicon energy barrier). A large drain voltage induces a lateral electric field (between source and drain). This field increases the energy of the electrons, thus making them hot. The hot electrons are injected from the channel into the floating gate or the charge-trapping layer, due to the vertical electric field applied from the gate. The injection mainly happens near the drain area, where the electrons have maximum energy. Electron is injected because it has sufficient energy higher than the barrier, which means that it should not suffer too many scattering events which reduce its energy while it moves from the source to the drain. Since the probability of the case is quite low, the electron, which does satisfy these conditions, is regarded as lucky. This also implies that hot electron injection is an inefficient method of programming since only a tiny part of channel electrons is injected. Figure 11 describes a schematic energy band diagram of it.

**Figure 12:**
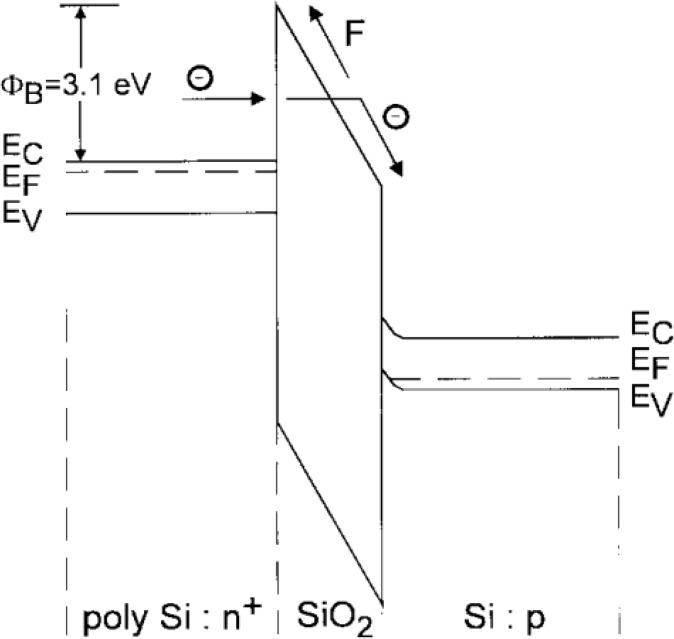
F-N tunneling of electrons from the poly-Si FG into the Si substrate through the triangular energy barrier posed by the tunneling layer. Reused with permission from [34], Copyright 1997, IEEE.

F-N tunneling refers to quantum-mechanical tunneling through a thin potential barrier, induced by an electric field. The oxide band bends under the application of an electric field, and presents a triangle-shaped barrier to the charge-trapping layer. The larger the electric field, the thinner the tunneling barrier is. Thus, more current occurs. F-N tunneling current is verified as a function of electric field and the current is exponentially dependent on the field. The probability of tunneling also depends on the distribution of carriers in the injecting material and the height of the barrier. Unlike the CHE discussed earlier, F-N tunneling injection is more efficient. Concerning the poor quality oxides that contain large numbers of bulk and interface traps, trap-assisted tunneling increases tunneling current density to much greater values, beyond expected, due to the effective decrease in the barrier. Figure 12 gives F-N tunneling of electrons from the poly-Si floating gate into the Si substrate. A table is also presented below (Table 4), including the advantages and disadvantages for the two charge injection mechanisms [34].

Originally, SiO_2_ is the tunneling layer in the SONOS structure on the Si substrate due to the largest band-gap among dielectrics, and, as the native oxide, the best interface quality as well as least oxide traps, thus lowest leakage current. For the consideration of reliability degradation issue, thermal oxide now is still dominant in charge trapping memory as tunneling layer. However, SONOS suffers from retention problems due to direct tunneling leakage through the thin tunnel oxide. For a conventional charge trapping memory device, it tends to have a thinner tunneling oxide for higher P/E speeds and lower operating voltages, whereas a thinner tunneling oxide may deteriorate its retention property.

**Figure 13:**
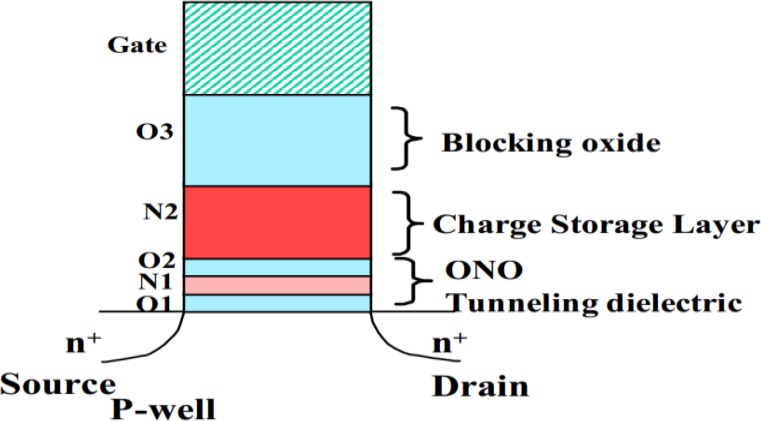
Structure of the n-channel BE-SONOS using ultra-thin ONO tunneling dielectric. Reused with permission from [55], Copyright 2003, IEEE.

A band-gap engineered ONO tunneling dielectric was proposed by Lue *et al.*, to replace the traditional tunnel oxide in SONOS, due to ultra-thin nitride (<2 nm) has negligible charge trapping [55]. This concept is demonstrated by a multilayer structure of O1/N1/O2/N2/O3, where the ultra-thin “O1/N1/O2” serves as a non-trapping tunneling dielectric, N2 the high-trapping-rate charge storage layer, and O3 the blocking oxide. The ultra-thin “O1/N1/O2” provides a “modulated tunneling barrier”. Structure of the device is shown in Figure 13. It suppresses direct tunneling at low electric field during retention, while it allows efficient hole tunneling erase at high electric field due to the band offset. The ONO tunneling dielectric serves as an efficient hole tunneling barrier for SONOS, and it is much more reliable and practical. Therefore, this SONOS offers fast hole tunneling erase, while it is immune to the retention problem of the conventional SONOS. The O1/N1/O2 tunneling dielectric consists of ultra-thin oxide (O1:1.5 nm, O2:1.8 nm) and nitride (2 nm). The thicker (7 nm) N2 serves as the charge-trapping layer and O3 (9 nm) serves as the blocking oxide. O3 is grown by thermal conversion of N2 so that efficient interfacial traps between N2/O3 are provided.

**Table 4:** Comparison between charge injection mechanisms [34].

	Channel Hot Electron (CHE)	Fowler-Nordheim (F-N)
**Advantages**	The physical mechanism of CHE is relatively simple to understand qualitatively. An electron traveling from the source to the drain gains energy from the lateral electric field and loses energy to the lattice vibrations. At low fields, this is a dynamic equilibrium condition, which holds until the field strength reaches approximately 100 kV/cm. For fields exceeding this value, electrons are no longer in equilibrium with the lattice, and their energy relative to the conduction band edge begins to increase. A lateral electric field (between source and drain) “heats” the electrons and a transversal electric field (between channel and control gate) injects the carriers through the oxide. The programming speed of CHE for conventional floating gate memory is faster.	The F-N mechanism is widely used in NVM, particularly in EEPROM. With a relatively thick oxide (20–30 nm) one must apply a high voltage (20–30 V) to have an appreciable tunnel current. With thin oxides, the same current can be obtained by applying a much lower voltage. An optimum thickness is chosen in present devices, which use the tunneling phenomenon to tradeoff between performance constraints (programming speed, power consumption, *etc.*) and reliability concerns. There are three main reasons for this choice: Tunneling is a pure electrical mechanism. The involved current level is quite low and thus allows the internal generation of supply voltages needed for all operations. It allows one to obtain the time to program (<1 ms) 12 orders of magnitude shorter than retention time (>10 *y*), which is a fundamental request for all NVM technologies.
**Disadvantages**	The probability of the injecting electrons is quite low and hot electron injection is an inefficient method of programming. For an electron to overcome the potential barrier, three requirements must be meet accordingly: The carrier has to be “lucky” enough to acquire enough energy from the lateral electric field to overcome the oxide barrier and to retain its energy after the collision that redirects the electron toward the interface *P*_Φb_. The carrier follows a collision-free path from the redirection point to the interface *P*_ED_. The carrier can surmount the repulsive oxide field at the injection point, due to the Schottky barrier lowering effect, without suffering an energy-robbing collision in the oxide *P*_OC_.	The exponential dependence of tunnel current on the oxide-electric field causes some critical problems of process control because. Very small variation of oxide thickness among the cells in a memory array produces a great difference in programming or erasing currents. Bad quality oxides are rich of interface and bulk traps, and trap-assisted tunneling is made possible since the equivalent barrier height seen by electrons is reduced and tunneling requires a much lower oxide field than 10 MV/cm. The oxide defects must be avoided to control program/erase characteristics and to have good reliability. Frequent program and erase operations induce an increase of trapped charge in the oxide. This affects the barrier height, which is lower in the case of positive and higher in the case of negative trapping, respectively, thus increasing or decreasing the tunnel currents.

A novel multilayer tunnel barrier concept was proposed consisting of a two-layer dielectrics stack with a low*-k*/high*-k* combination (or three-layer stack with a low*-k*/high*-k*/low*-k* sequence in its symmetric form) that allows for either lower voltage or higher speed programming due to the increased current–voltage (I–V) slope [56]. Furthermore, its implementation is compatible with the high*-k* dielectrics considered for SiO_2_ replacement in sub-100 nm CMOS technologies and represents a viable alternative for low-voltage low-power sub-100 nm NVM nodes. A barrier thinning of the stack was noticed and, in this sense, the stack can be regarded as a VARIable Oxide Thickness (VARIOT) dielectric. Alternatively, lower voltage programming at identical speed is possible. The thicker physical thickness of the stack offers better retention as compared to the EOT layer at low biases. An 8 nm SiO_2_ (assumed as low*-k*) layer would satisfy the retention requirements for biases up to 3.6 V, whereas FN programming in 100 μs would be achieved at nearly 10 V. For 5 nm SiO_2_ thickness, the programming bias decreases to 5.6 V, whereas the maximum bias during retention decreases to nearly 1.5 V. By contrast, in a two-layer SiO_2_/Al_2_O_3_ stack of the same EOT and with 2 nm SiO_2_, the retention condition is satisfied for stack biases as high as 3 V, whereas programming in 100 μs can be achieved at virtually the same bias as for the EOT layer. A symmetric structure with the same material combination (1.6 nm SiO_2_ on each side) merely results in a slight performance decrease, allowing for both program and erase operations at comparable speeds. If voltage decrease is the prime issue, a two-layer stack (e.g., SiO_2_/ZrO_2_ with 1 nm SiO_2_) with the second layer having an even higher *k* value lows for very low-voltage programming. Therefore, it is concluded that for the same voltage, a shorter tunnel path of charges from the substrate to the charge-trapping layer is able to be obtained for the structure. Alternatively, a lower operating voltage can be achieved for the structure in order to obtain the same electric field across the tunneling layer as for the SiO_2_ structure.

## 4. Conclusions

Flash memories are ubiquitous in their use as portable storage media in cell phones, cameras, music players, and other portable electronic devices. Flash memory device, consisting of a charge-trapping transistor cell, is the most aggressively scaled electronic device, as evidenced by ever-increasing memory capacities. In this paper, we examine possible problems arising from continued scaling of these structures, and discuss novel solutions with high*-k* dielectric to overcome them. Combining ultra-thin tunneling layer, high*-k* dielectric charge-trapping layer, and large barrier height blocking layer, we enable the updated charge trapping memory cell as a scalable candidate for the next-generation technology. Every potential candidate has its own features and it might improve the related specification for charge trapping memory’s performance at the cost of other issues, like fabrication technique, reliability degradation, scaling limitation or mass production cost. In general, some of the future research direction might be investigated. Tunnel oxide thickness scaling has essentially stopped. Work on barrier-engineered layers is an important area that has potential. The process steps involved in the planar floating-gate device need to be further optimized. For example, there needs to be better understanding for deposition of high quality high-*k* dielectrics on metals, and interaction of the metal-tunnel oxide interface. As the cells become smaller and smaller, they will be limited by the number of electrons stored. At that point, instead of scaling cell-area, it is better to integrate cells in 3D to increase density. Work towards this, like the bit cost scalable (BiCS) cell, is an important direction of research. At those ultra-small dimensions, as the flash cell becomes intrinsically limited, some advanced non-charge based cells like FeRAM, MRAM, PCRAM, and ReRAM are also regarded as promising alternatives.
